# Targeted AntiBiotics for Chronic pulmonary diseases (TARGET ABC): can targeted antibiotic therapy improve the prognosis of *Pseudomonas aeruginosa*-infected patients with chronic pulmonary obstructive disease, non-cystic fibrosis bronchiectasis, and asthma? A multicenter, randomized, controlled, open-label trial

**DOI:** 10.1186/s13063-022-06720-z

**Published:** 2022-09-27

**Authors:** Josefin Eklöf, Imane Achir Alispahic, Pradeesh Sivapalan, Torgny Wilcke, Niels Seersholm, Karin Armbruster, Jakob Lyngby Kjærgaard, Mohamad Isam Saeed, Thyge Lynghøj Nielsen, Andrea Browatzki, Rikke Holmen Overgaard, Camilla Sund Fenlev, Zitta Barella Harboe, Helle Frost Andreassen, Therese Sophie Lapperre, Lars Pedersen, Stine Johnsen, Charlotte Suppli Ulrik, Julie Janner, Mia Moberg, Maria Heidemann, Ulla Møller Weinreich, Roxana Vijdea, Hans Linde, Ingrid Titlestad, Sofie Lock Johansson, Flemming Schønning Rosenvinge, Christian Østergaard, Khaled Saoud Ali Ghathian, Lise Gundersen, Christina Wellendorph Christensen, Jette Bangsborg, Torben Tranborg Jensen, Vibeke Muff Sørensen, Thilde Ellingsgaard, Raluca Datcu, John Eugenio Coia, Uffe Bodtger, Jens Ulrik Stæhr Jensen

**Affiliations:** 1 Department of Internal Medicine, Section of Respiratory Medicine, Herlev and Gentofte Hospital, University of Copenhagen, Copenhagen, Denmark; 2 Department of Internal Medicine, Zealand Hospital, University of Copenhagen, Roskilde, Denmark; 3 Department of Respiratory and Infectious Diseases, Frederikssund and Hillerød Hospital, University of Copenhagen, Copenhagen, Denmark; 4 Department of Respiratory Medicine, Bispebjerg Hospital, University of Copenhagen, Copenhagen, Denmark; 5 Department of Respiratory Medicine, Amager and Hvidovre Hospital, University of Copenhagen, Copenhagen, Denmark; 6 Department of Respiratory Medicine, Aalborg University Hospital, University of Aalborg, Aalborg, Denmark; 7 Department of Clinical Microbiology, Aalborg University Hospital, University of Aalborg, Aalborg, Denmark; 8 Department of Respiratory Medicine, Odense University Hospital, University of Southern Denmark, Odense, Denmark; 9 Department of Clinical Microbiology, Odense University Hospital, University of Southern Denmark, Odense, Denmark; 10 Department of Clinical Microbiology, Amager and Hvidovre Hospital, University of Copenhagen, Copenhagen, Denmark; 11 Department of Clinical Microbiology, Herlev and Gentofte Hospital, University of Copenhagen, Copenhagen, Denmark; 12 Department of Internal Medicine, Section of Respiratory Medicine, Hospital of South West Jutland, Esbjerg, Denmark; 13 Department of Clinical Microbiology, Hospital of South West Jutland, Esbjerg, Denmark; 14 Department of Respiratory Medicine, Naestved Hospital, University of Southern Denmark, Naestved, Denmark; 15 PERSIMUNE: Department of Infectious Diseases, Rigshospitalet, University of Copenhagen, Copenhagen, Denmark

**Keywords:** Chronic obstructive pulmonary disease, Non-CF bronchiectasis, Asthma, *Pseudomonas aeruginosa*, Antibiotics, Randomized controlled trial

## Abstract

**Background:**

*Pseudomonas aeruginosa* infection is seen in chronic pulmonary disease and is associated with exacerbations and poor long-term prognosis. However, evidence-based guidelines for the management and treatment of *P. aeruginosa* infection in chronic, non-cystic fibrosis (CF) pulmonary disease are lacking. The aim of this study is to investigate whether targeted antibiotic treatment against *P. aeruginosa* can reduce exacerbations and mortality in patients with chronic obstructive pulmonary disease (COPD), non-CF bronchiectasis, and asthma.

**Methods:**

This study is an ongoing multicenter, randomized, controlled, open-label trial. A total of 150 patients with COPD, non-CF bronchiectasis or asthma, and *P. aeruginosa*-positive lower respiratory tract samples will be randomly assigned with a 1:1 ratio to either no antibiotic treatment or anti-pseudomonal antibiotic treatment with intravenous beta-lactam and oral ciprofloxacin for 14 days. The primary outcome, analyzed with two co-primary endpoints, is (i) time to prednisolone and/or antibiotic requiring exacerbation or death, in the primary or secondary health sector, within days 20–365 from study allocation and (ii) days alive and without exacerbation within days 20–365 from the study allocation.

**Discussion:**

This trial will determine whether targeted antibiotics can benefit future patients with chronic, non-CF pulmonary disease and *P. aeruginosa* infection in terms of reduced morbidity and mortality, thus optimizing therapeutic approaches in this large group of chronic patients.

**Trial registration:**

ClinicalTrials.gov NCT03262142. Registered on August 25, 2017.

**Supplementary Information:**

The online version contains supplementary material available at 10.1186/s13063-022-06720-z.

## Administrative information

**Table:** 

Title {1}	Targeted AntiBiotics for Chronic pulmonary diseases (TARGET ABC): can targeted antibiotic therapy improve the prognosis of *Pseudomonas aeruginosa*-infected patients with chronic pulmonary obstructive disease, non-cystic fibrosis bronchiectasis, and asthma? A multicenter, randomized, controlled, open-label trial
Trial registration {2a and 2b}	TRLS-D-21-00986
Protocol version {3}	Version 2
Funding {4}	Danmarks Frie Forskningsfond (8020-00425B)
Author details {5a}	Josefin Eklöf, Imane Achir Alispahic, Pradeesh Sivapalan, Torgny Wilcke, Niels Seersholm, Karin Armbruster, Jakob Lyngby Kjærgaard, Mohamad Isam Saeed, Thyge Lynghøj Nielsen, Andrea Browatzki, Rikke Holmen Overgaard, Camilla Sund Fenlev, Zitta Barella Harboe, Helle Frost Andreassen, Therese Sophie Lapperre, Lars Pedersen, Stine Johnsen, Charlotte Suppli Ulrik, Julie Janner, Mia Moberg, Maria Heidemann, Ulla Møller Weinreich, Roxana Vijdea, Hans Linde, Ingrid Titlestad, Sofie Lock Johansson, Flemming Schønning Rosenvinge, Christian Østergaard, Khaled Saoud Ali Ghathian, Lise Gundersen, Christina Wellendorph Christensen, Jette Bangsborg, Torben Tranborg Jensen, Vibeke Muff Sørensen, Thilde Ellingsgaard, Raluca Datcu, John Eugenio Coia, Uffe Bodtger, Jens-Ulrik Stæhr Jensen
Name and contact information for the trial sponsor {5b}	Jens Ulrik Stæhr Jensen, MD, PhD, Professor jens.ulrik.jensen@regionh.dk
Role of sponsor {5c}	Scientific sponsor. Applying for financial research grant. Coordinator for the COP:TRIN steering committee.

## Introduction

### Background and rationale

COPD, non-CF bronchiectasis, and asthma are common chronic pulmonary diseases and important causes of death and disability worldwide [1–3]. These diseases are characterized by shared common symptoms such as productive cough and susceptibility to recurrent exacerbations that are often associated with infections. These exacerbations lead to accelerated loss of lung function, reduced quality of life, and increased morbidity and mortality and have major socio-economic consequences [4–6].

Compared to COPD and asthma, which both are diagnosed on the basis of airflow obstruction and therefore are physiological diagnoses, bronchiectasis is a structural diagnosis with the presence of permanent airway dilatation on radiological imaging [4, 6, 7]. However, the co-existence of bronchiectasis and asthma or COPD is common [8].

*Pseudomonas aeruginosa* [9] has been reported to be present in the lower airways in up to 20% of patients with COPD [10–12] and is frequently detected in patients with non-CF bronchiectasis [13]. The bacterium has also been observed in patients with asthma [14]. Nevertheless, the influence of *P. aeruginosa* on the progression of these diseases is far from fully elucidated. The bacterium is seen primarily in advanced diseases with severely impaired lung function [13–15] and is associated with increased frequency of exacerbation, prolonged hospitalization, and poor long-term prognosis with increased mortality rates compared to *P. aeruginosa*-negative patients [16].

However, since an impairment of lung function itself is a strong predictor of morbidity and mortality, it is not certain whether infection with *P. aeruginosa* is secondary to lung function impairment or whether the presence of *P. aeruginosa* itself leads to pulmonary tissue inflammation and remodeling, impaired lung function, and overall poor prognosis.

Thus, the role of *P. aeruginosa* on the progression of COPD, non-CF bronchiectasis, and asthma is poorly characterized. To date, evidence-based guidelines for the management and treatment of *P. aeruginosa* infection are lacking, and the management of *P. aeruginosa* is often based on expert consensus and studies of other chronic lung diseases, including CF. In CF, *P. aeruginosa* is a leading cause of morbidity and early death with evidence of improved clinical outcomes through aggressive and targeted antibiotic treatment [17]. In Denmark, the first treatment choice for clinically treatment-requiring *P. aeruginosa* infection is usually 10–14 days of antibiotic combination therapy with intravenous piperacillin/tazobactam and oral ciprofloxacin [18].

The SPIRIT reporting guidelines have been used for the present protocol [18].

### Objectives

With this randomized controlled trial, we aim to increase the understanding of the clinical significance and consequences of *P. aeruginosa* infection in patients with chronic, non-CF pulmonary disease.

The main purpose is to investigate if targeted, antibiotic treatment of *P. aeruginosa* improves the disease prognosis in patients with exacerbation of COPD, non-CF bronchiectasis or asthma, and *P. aeruginosa*-positive sputum/bronchoalveolar lavage (BAL) sample.

Our hypothesis is that antipseudomonal antibiotics given for 14 days increase the number of days alive and out of hospital for 1 year*.*

### Trial design

The study is a multicenter, randomized, controlled, open-label trial in patients with COPD, non-CF bronchiectasis, or asthma with current *P. aeruginosa*-positive lower respiratory tract sample. Study participants are followed for 1 year.

## Methods: participants, interventions, and outcomes

### Study setting

**Fig. 1:**
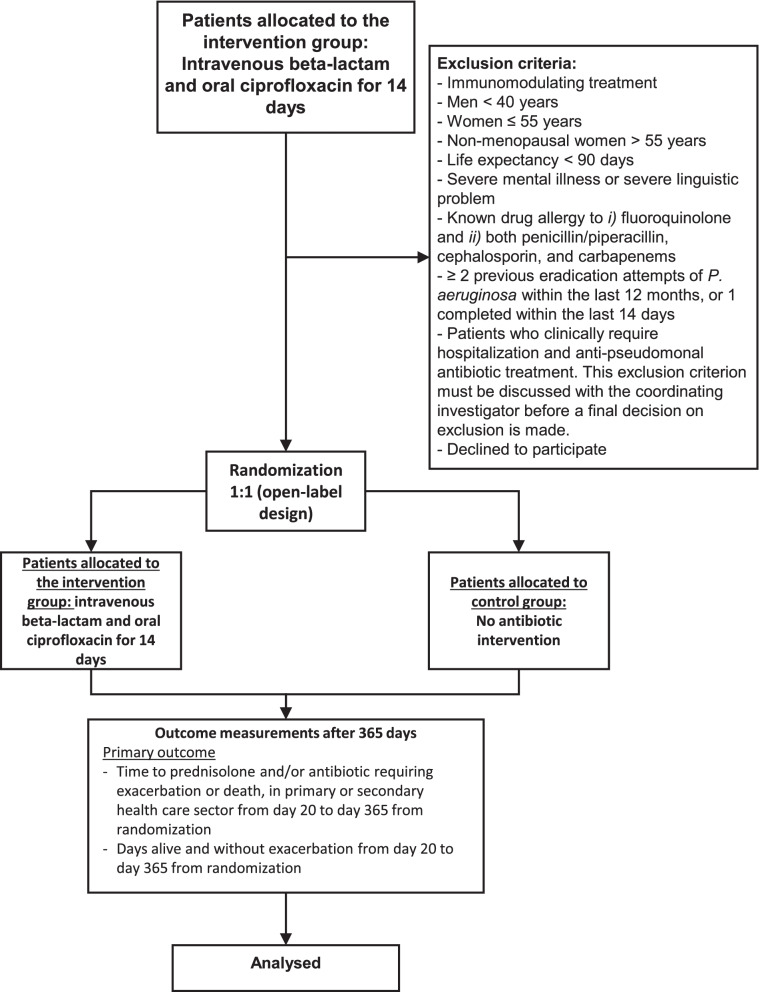
Flow diagram for the Targeted AntiBiotics for COPD trial: primary outcome overview

Participants are recruited by investigators who are employed at the participating pulmonary departments in Denmark. In total, 150 patients are expected to be included in the study (Fig. 1). The sample size is calculated based on a superiority framework with a two-sided 5% significance level 80% power and the following estimates and indicative figures. They are enrolled in seven different sites: Sydvestjysk Hospital, Aarhus University Hospital, Bispebjerg Hospital, Herlev and Gentofte Hospital, Hvidovre Hospital, Nordsjællansk Hospital, and Odense University Hospital.

### Eligibility criteria

Patients with COPD, non-CF bronchiectasis or asthma, and current *P. aeruginosa*-positive lower respiratory tract samples (i.e., sputum, tracheal secretion, bronchial secretion, and bronchial alveolar lavage) from the participating study centers are considered for study enrollment. Patients are invited to participate in the trial if they fulfill the following inclusion and exclusion criteria.

The following are the inclusion criteria:

1. *P. aeruginosa*-positive lower respiratory tract sample
2. COPD, non-CF bronchiectasis, or asthma verified by a respiratory specialist based on clinical assessment and additional tests:
  - COPD: spirometry
  - Asthma: reversibility
  - Non-CF bronchiectasis: high-resolution computed tomography scan
3. Minimum of two previous exacerbations, or one previous hospitalization-requiring or emergency room-demanding exacerbation, with the treatment of systemic prednisolone and/or antibiotics within the last 12 months
4. Written informed consent

**Fig. 2:**
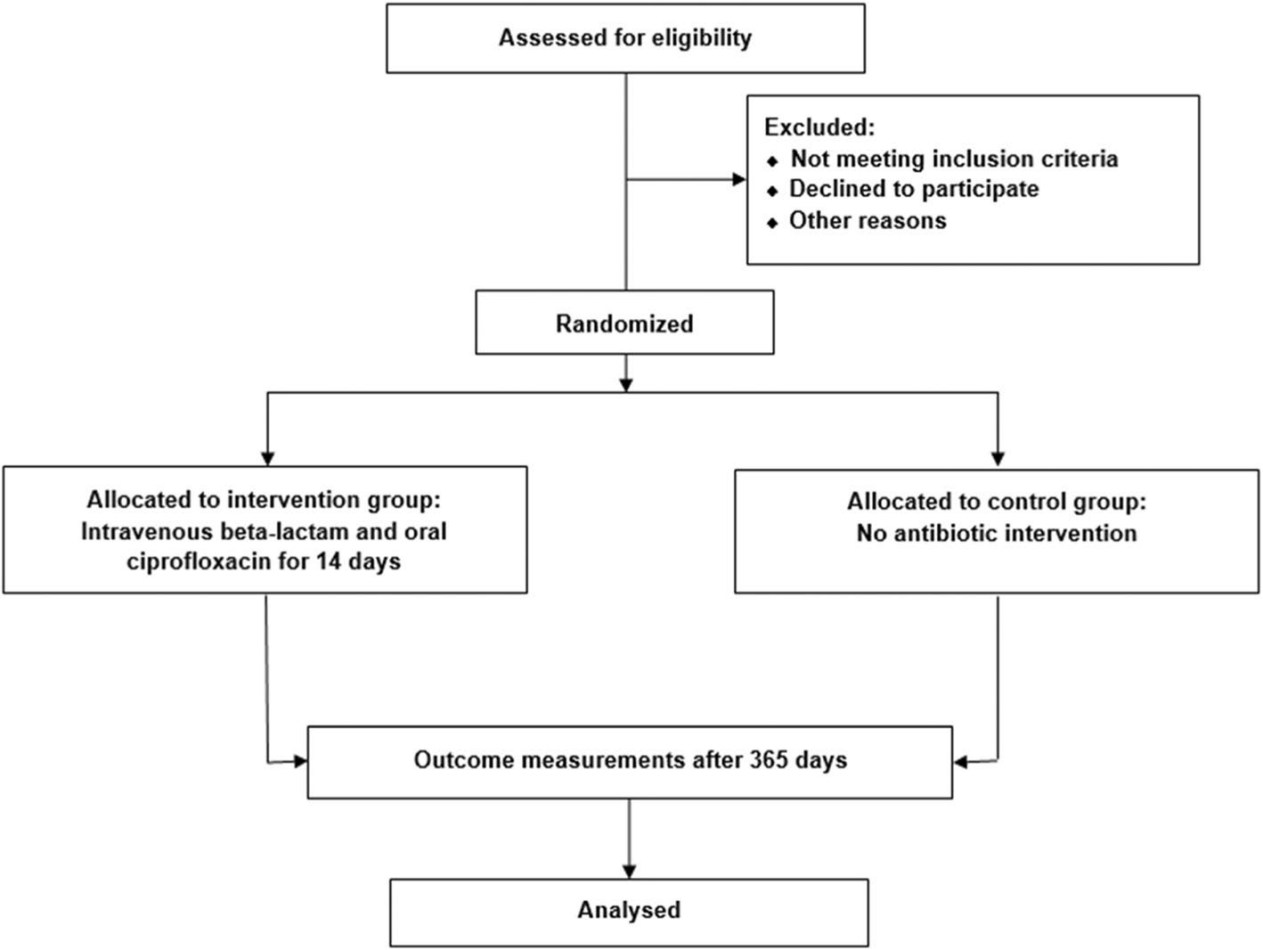
CONSORT flow diagram for the Targeted AntiBiotics for COPD trial

The following are the exclusion criteria:

1. Immunomodulating treatment (except ≤ 10 mg prednisolone/day)
2. Men < 40 years
3. Women ≤ 55 years
4. Non-menopausal women > 55 years (i.e., menstruation within the last 12 months)
5. Life expectancy < 90 days
6. Severe mental illness or severe linguistic problem
7. Known drug allergy to (i) fluroquinolone and (ii) both penicillin/piperacillin, cephalosporin, and carbapenems
8. ≥ 2 previous eradication attempts of *P. aeruginosa* within the last 12 months or 1 completed within the last 14 days
9. Patients who clinically require hospitalization and anti-pseudomonal antibiotic treatment. This exclusion criterion must be discussed with the coordinating investigator before the final decision on exclusion is made (Fig. 2).

### Project management

The study is managed by the coordinating investigator (Josefin Eklöf). The daily project management is carried out by sub-investigators, consisting of health professionals from the departments involved in the trial. A filled patient informed consent form is required from all participants in the study and must be signed by both the study participant and the informing investigator. A separate consent form is obtained from the participants to store the whole blood and serum in a biobank.

### Intervention

Patients will be randomly assigned 1:1 to either of the following:

I. Control group: no antibiotic treatment
II. Intervention group: antibiotic treatment

The first choice of antibiotic treatment is dual therapy with intravenous piperacillin/tazobactam 4/0.5 g, 4 times daily, and oral ciprofloxacin 500 mg, 2 times daily for 14 days. In case of penicillin allergy or antibiotic resistance, intravenous piperacillin/tazobactam is replaced by intravenous ceftazidime or meropenem. In case of fluoroquinolone allergy or antibiotic resistance, intravenous beta-lactam is given as monotherapy.

### Criteria for discontinuing or modifying allocated interventions

Sub-investigators may interrupt the intervention at any time if there is a medical justification, safety risk, or a requirement from the authorities. If the investigator deems it necessary, he or she may exclude the participant from the trial. However, in general, no subject should be removed from the study for a protocol violation prior to confirmation by the coordinating investigator. In addition, a study participant can at any time withdraw from the study without the needed explanation.

### Strategies to improve adherence to interventions

The participants are well-informed at randomization about the importance of following our guidance. The patients, who received treatment, are hospitalized in order to give them intravenous antibiotics. This ensures that the patients take their medicine at the right time.

### Outcomes

The primary outcomes, analyzed with two co-primary endpoints, are as follows:

- Time to prednisolone and/or antibiotic requiring exacerbation or death, in primary or secondary health care sectors from day 20 to day 365 from randomization
- Days alive and without exacerbation from day 20 to day 365 from randomization

The secondary endpoints are as follows:

- Death within 365 days from randomization
- Number of re-admissions with pulmonary exacerbation within 365 days from randomization
- Number of days with non-invasive ventilation or invasive ventilation within 90 days from randomization
- Microbiological cure*
- Clinical cure day 14**
- Change in COPD Assessment Test (CAT) from randomization to day 90
- Change in body mass index (BMI) from randomization to day 90
- Change in forced expiratory volume in the first second (FEV_1_) from randomization to day 90
- Decrease of ≥ 200 ml in FEV_1_ from randomization to day 365

*Microbiological cure: *P. aeruginosa*-negative sputum culture until day 90. No microbiological cure: positive sputum culture with clonally same *P. aeruginosa* strain ≤ day 90. Re-infection: positive sputum sample with non-clonally same *P. aeruginosa* strain ≤ day 90.

**Clinical cure: improvement of clinical signs and symptoms related to *P. aeruginosa* before or on day 14. Clinical failure: persistent or worsening of clinical signs and symptoms related to *P. aeruginosa* before or on day 14.

### Sample size

Data will be analyzed using intention-to-treat (ITT) principles, including all the data available, regardless of whether the intervention was completed or not. The aim of the ITT analysis is also to provide unbiased comparisons among the two study groups and to avoid the effects of potential study dropouts and protocol deviations.

Patients in the control group will be compared to patients in the intervention group. We will use Fisher’s exact test and chi-squared test for dichotomous outcomes and *T*-test for continuous outcomes. The timed dichotomous outcomes will be visualized through Kaplan-Meier plots. Furthermore, adjusted analyses will be performed with a multivariable Cox proportional hazards model, adjusting for baseline variables and calculating hazard ratios. Data will be processed and analyzed in SAS and graphs are generated in Microsoft Excel and other graph programs.

COPD background:

1. *P. aeruginosa* incidence 5–20%
2. Sixty-seven percent of study participants in the antibiotic-free group have exacerbated or died within 12 months
3. Forty-seven percent of study participants in the antibiotic group have exacerbated or died within 12 months

Thus, we expect an effect size of 20% absolute reduction (30% relative reduction) of exacerbation or mortality in the antibiotic group. Based on the above, a total of 150 patients should be included (i.e., 75 patients in each group). Furthermore, to avoid error estimates and risk of including too few patients (underpowering), the study is “event-driven” and will only be closed when at least 67% of patients in the antibiotic-free group have exacerbated or died, but 12 months must have passed, also if > 67% of patients have experienced the primary outcome event.

Bronchiectasis or asthma background:

1. Annual exacerbation rate with *P. aeruginosa*: 2.85
2. Annual exacerbation rate without *P. aeruginosa*: 1.80
3. Standard deviation, annual exacerbation rate: 1.5

Based on these reference estimates [19], a total of 66 patients should be included (i.e., 33 patients in each study group).

Since most study participants potentially could be COPD patients, the number from the COPD sample size calculation is used in order not to risk underpowering the study. If non-COPD patients are recruited, the power will thus be increased to > 80%.

In cases of low *P. aeruginosa* incidence (PAi), patients will need to be recruited from several pulmonary departments to achieve the desired sample size, as calculated below. The figures are based on the estimation of approximately 2000 patients with COPD class C/D/outpatient clinic. Of these, 1/3 are expected to be able to produce sputum samples. However, the dropout rate is estimated to be about the same level; thus, there are approximately potentially 400 patients/outpatient clinic/year.

$${\displaystyle \begin{array}{c}5\%\mathrm{PAi}:0.05\times 400=20\ \mathrm{patients}/\mathrm{outpatient}\ \mathrm{clinic}/\mathrm{year}\times 8\ \mathrm{outpatient}\ \mathrm{clinic}\mathrm{s}=160\ \mathrm{patients}/\mathrm{year}\\ {}10\%\mathrm{PAi}:0.10\times 400=40\ \mathrm{patients}/\mathrm{outpatient}\ \mathrm{clinic}/\mathrm{year}\times 4\ \mathrm{outpatient}\ \mathrm{clinic}\mathrm{s}=160\ \mathrm{patients}/\mathrm{year}\\ {}15\%\mathrm{PAi}:0.15\times 400=60\ \mathrm{patients}/\mathrm{outpatient}\ \mathrm{clinic}/\mathrm{year}\times 3\ \mathrm{outpatient}\ \mathrm{clinic}\mathrm{s}=180\ \mathrm{patients}/\mathrm{year}\\ {}20\%\mathrm{PAi}:0.20\times 400=80\ \mathrm{patients}/\mathrm{outpatient}\ \mathrm{clinic}/\mathrm{year}\times 2\ \mathrm{outpatient}\ \mathrm{clinic}\mathrm{s}=160\ \mathrm{patients}/\mathrm{year}\end{array}}$$

### Assignment of interventions: allocation

#### Sequence generation

Pre-stratified block randomization with blocks of varying and blinded size is applied to ensure equal distribution of patients in the study groups based on site (pulmonary department) and age (above or below 70 years of age). The allocation sequence is generated by Pradeesh Sivapalan, with instuctions from the scientific sponsor of the trial. The final randomization is conducted using a secure web application (REDCap; www.projectredcap.org) where inclusion and exclusion criteria are required to be filled out correctly in order to randomize a study participant. Each investigator had access to allocation and randomization through REDCap.

#### Assignment of interventions: blinding

Our study is not blinded. It is an open-label trial. Home treatment with antibiotics was not feasible due to local restrictions at the participating study sites. Thus, attempting blinding would demand hospital admission of control patients to receive placebo infusions. Sites expressed that they would not participate with such a design, so the Steering Committee found it non-feasible. Moreover, we anticipated that a blinded placebo intervention would be a major barrier to recruiting patients.

### Data collection and management

#### Plans for assessment and collection of outcomes

Data is collected in case report forms, specific to each participant, where demographic data, current and past illnesses, health status, hospitalizations, clinical parameters, study results, and prescribed medication are recorded. Follow-up visits are scheduled after 14, 30, 60, 90, and 365 days. The study overview is summarized in Table 1. Antibiotic therapy is administered according to standard practice, including routine adjustment based on co-medication, kidney function, age, etc. The administration of antibiotics is registered using the standard electronic medicine module in the department. High-resolution computed tomography is performed at the start of the study to identify the underlying presence of emphysema and bronchiectasis.

**Table 1:** SPIRIT figure illustrating the TARGET ABC study overview

Study period							
	Enrollment	Intervention	Follow-up				
**Visit number**	**1**		**2**	**3**	**4**	**5**	**6**
**Study day**	**0**	**0–14**	**14**	**30**	**60**	**90**	**365**
**Enrollment**							
Eligibility screening	X						
Informed consent	X						
Randomization	X						
**Study arm**							
Intervention group: antibiotic treatment, in-hospital		X					
Control group: no antibiotic treatment, not hospitalized							
**Data collection and examinations**							
Demographics	X						
Sputum sample	X		X	X	X	X	X
Body mass index (BMI)	X		X	X	X	X	X
Medical Research Council Dyspnea Scale (MRC)	X		X	X	X	X	X
COPD Assessment Test (CAT)	X		X	X	X	X	X
Spirometry	X		X	X	X	X	X
Vital parameters	X		X				
High-resolution computed tomography (HRCT)	X						
Blood samples	X	X	X				

#### Plans to promote participant retention and complete follow-up

At the inclusion date, every patient is well-informed about the number of visits, so the patient knows exactly what she/he is going into. The patient and investigator agree on the next visit date at every follow-up visit.

#### Confidentiality

The collection of data and storage is in compliance with the Good Clinical Practice (GCP) guidelines and is regularly monitored by local GCP units. Data is stored in a double-locked locker, which only sub-, coordinating, and principal investigators have access to.

#### Plans for collection, laboratory evaluation, and storage of biological specimens for genetic or molecular analysis in this trial/future use

A research biobank is set up with whole blood from the time of randomization and serum, EDTA plasma, and citrate plasma from the time of randomization and day 14 for future unspecified research. Sputum samples are longitudinally collected from the time of randomization and days 14, 30, 60, 90, and 365 for future microbiome and genetic analyses.

## Statistical methods

### Statistical methods for primary and secondary outcomes

A detailed statistical analysis plan is made and is attached.

### Oversight and monitoring

#### Composition of the coordinating center and trial steering committee

The coordinating center is Gentofte Hospital, where the principal investigator is located. The seven sites are weekly in contact with the coordinating investigator through online meetings. The trial steering committee is driven by professors and senior consultants from different sites. The trial steering committee is invited to a meeting twice a year. It is the coordinating site that decides if more meetings are needed.

#### Composition of the data monitoring committee, its role, and reporting structure

The data monitoring committee, which is also known as the GCP units, closely monitors every site (several times per year and per site). Their role is to check every included patient at every site and to see if the protocol has been followed, and the registrations are correctly made. After a GCP unit has checked the site, a report is then made and sent to the sponsor, the coordinating investigator, and the sub-investigators responsible for the site.

#### Adverse event reporting and harms

The occurrence of adverse events and adverse effects will be registered and reported to the Danish Medicines Agency at the end of the trial. Moreover, any serious adverse events and adverse effects are reported annually in a safety report to the Danish Medicines Agency.

#### Plans for communicating important protocol amendments to relevant parties (e.g., trial participants, ethical committees)

If any changes are made, every site will be informed promptly by mail and through Trial Setting Committee meetings.

#### Dissemination plans

The data from the TARGET ABC trial will be available once the study is completed. All results will be published in scientific contexts, including international journals, regardless of whether they are positive, negative, or inconclusive, and with authorship according to the Vancouver recommendations.

## Discussion

*P. aeruginosa* represents a potentially significant cause of exacerbation and mortality in patients with COPD, non-CF bronchiectasis, and asthma. However, the influence of *P. aeruginosa* on the progression of these chronic pulmonary diseases is poorly characterized, and to date, evidence-based guidelines for the management and treatment of *P. aeruginosa* infection are lacking. With this trial, we aim to increase the understanding of the clinical significance and consequences of *P. aeruginosa* infection in patients with non-CF chronic pulmonary disease.

Using a multicenter, randomized, controlled design, we will allocate 150 patients with COPD, non-CF bronchiectasis, or asthma and a current *P. aeruginosa*-positive sputum/BAL sample evenly to either no antibiotic treatment or 14 days of dual anti-pseudomonal antibiotic therapy. Thus, we seek to create evidence at level 1B to determine whether targeted antibiotic treatment against *P. aeruginosa* can improve the prognostic outcome in this large group of patients with chronic pulmonary disease.

The trial is carried out according to the Declaration of Helsinki and in accordance with the Good Clinical Practice guidelines. The study methods and statistical analyses have been carefully considered, and it is our strongest belief that the trial will contribute essential knowledge that will help clinicians to guide future patients towards evidence-based and improved treatment strategies, ultimately improving the disease prognosis.

### Trial status

Patient recruitment commenced in October 2017 and is ongoing.

## Supplementary Information

**Additional file 1.**

**Additional file 2.**

## Data Availability

The data from the TARGET ABC study will be available once the study is completed. Applications for data require a formal application and will be decided upon by the board of the TARGET ABC study group.

## Abbreviations

BAL: Bronchoalveolar lavage
BMI: Body mass index
CAT: COPD assessment test
CF: Cystic fibrosis
COPD: Chronic obstructive pulmonary disease
CT: Computed tomography
FEV_1_: Forced expiratory volume in the first second
GCP: Good Clinical Practice
HRCT: High-resolution computed tomography
MRC: Medical Research Council
PAi: *Pseudomonas aeruginosa* incidence
