# Supplementary material for: Targeted AntiBiotics for Chronic pulmonary diseases (TARGET ABC): can targeted antibiotic therapy improve the prognosis of Pseudomonas aeruginosa-infected patients with chronic pulmonary obstructive disease, non-cystic fibrosis bronchiectasis, and asthma? A multicenter, randomized, controlled, open-label trial
Source: Trials. 2022 Sep 27;23:817. doi: 10.1186/s13063-022-06720-z (PMC9513970; doi:10.1186/s13063-022-06720-z)
Supplement: Supplementary file 1 — Additional file 1. [file 13063_2022_6720_MOESM1_ESM.docx]

Appendix

Informed consent form for participation in a health science research project:

**Targeted AntiBiotics for Chronic pulmonary diseases (TARGET ABC).**

**Can targeted antibiotic therapy improve the prognosis of patients with chronic pulmonary obstructive disease, non-cystic fibrosis bronchiectasis, and asthma infected with *Pseudomonas aeruginosa*?**

**An “open-label” randomized multicenter study.**

**Statement from the participant:**

I have been given written and oral information and I know enough about the purpose, method, benefits, and disadvantages to say yes to attend.

I know that participating is voluntary and that I can always withdraw my consent without losing my current or future rights to treatment.

I agree to participate in the research project and to have my biological material extracted for the purpose of storage in a current research biobank as well as a biobank for future research. I have received a copy of this consent sheet as well as a copy of the written information about the project for my own use.

Name of participant: ________________________________________________________

Date: _______________ Signature: ____________________________________________

If any new essential health information about you appears in the research project you will be informed. If you will **decline** information about new significant health information that will appear in the research project, please mark here: __________ (mark with X)

Do you want to be informed about the results of the research project and any eventual consequences for you?

Yes _____ (mark with X) No _____ (mark with X)

**Declaration by the provider of information:**

I declare that the participant has received oral and written information about the trial.

In my opinion, sufficient information has been provided to enable study participation in the trial.

Name of the provider of information: ______________________________________________________

Date: _______________ Signature: ____________________________________________

**Informed consent for the storage of blood and saliva samples for future research**

**Targeted AntiBiotics for Chronic pulmonary diseases (TARGET ABC).**

**Can targeted antibiotic therapy improve the prognosis of patients with chronic pulmonary obstructive disease, non-cystic fibrosis bronchiectasis, and asthma infected with *Pseudomonas aeruginosa*?**

**An “open-label” randomized multicenter study.**

**Statement from the participant:**

• I have been given written and verbal information about the rationale for storing blood and saliva samples for future research.

• I know that it is voluntary and that I can always withdraw my consent without losing my current or future rights to processing.

• I give consent for the samples to be taken with respect to storage in a research biobank for later scientific use.

Subject’s name:_________________________________________________

Date: Signature: __________________

**Declaration by the person providing the information:**

I declare that the subject has received verbal and written information about the collection and storage of biological material for future research.

In my opinion, sufficient information has been provided for a decision to be made regarding consent to the above.

The name of the person who submitted the information:________________________________

Date: Signature:
