# Supplementary material for: Targeted AntiBiotics for Chronic pulmonary diseases (TARGET ABC): can targeted antibiotic therapy improve the prognosis of Pseudomonas aeruginosa-infected patients with chronic pulmonary obstructive disease, non-cystic fibrosis bronchiectasis, and asthma? A multicenter, randomized, controlled, open-label trial
Source: Trials. 2022 Sep 27;23:817. doi: 10.1186/s13063-022-06720-z (PMC9513970; doi:10.1186/s13063-022-06720-z)
Supplement: Supplementary file 2 — Additional file 2. [file 13063_2022_6720_MOESM2_ESM.docx]

Version 1 17 august 2022

**Statistical Analysis Plan**

**Targeted AntiBiotics for Chronic pulmonary diseases (TARGET-ABC)**

**A multicenter, randomized, controlled, open-label trial**

**ClinicalTrials.org Identifier:** NCT03262142

**Author:** Josefin Eklöf

**Introduction:**

This is a multicenter, randomized, controlled, open-label trial evaluating the effect of antibiotic treatment for *P. aeruginosa* in patients with chronic pulmonary disease.

The aim of the study is to investigate whether targeted antibiotics against *P. aeruginosa* can reduce exacerbations and mortality in patients with chronic obstructive pulmonary disease (COPD), non-CF bronchiectasis (non-CF BE) and asthma.

The patients are enrolled in the trial only after obtaining informed consent. The trial is conducted at seven centers in Denmark:

**1.** Department of Internal Medicine, Section of Respiratory Medicine, Herlev-Gentofte Hospital, University of Copenhagen.

Primary investigator: Josefin Eklöf, MD, PhD.

**2**. Department of Pulmonary and Infectious Diseases, University Hospital North Zealand Hospital. Primary investigator: Andrea Browatzki, MD.

**3.** Department of Respiratory Medicine, Amager-Hvidovre Hospital, University of Copenhagen.

Primary investigator: Julie Janner, MD, PhD.

**4.** Department of Respiratory Medicine, Bispebjerg-Frederiksberg Hospital, University of Copenhagen.

Primary investigator: Therese Lapperre, MD, PhD, Research Associate Professor.

**5.** Department of Respiratory Medicine, Aalborg Hospital, University of Aalborg.

Primary investigator: Ulla Weinreich, MD, PhD, Research Associate Professor.

**6.** Department of Respiratory Medicine, Odense University Hospital.

Primary investigator: Sofie Johansson, MD, PhD

**7.** Department of Internal Medicine, Section of Respiratory Medicine, Hospital of South West Jutland, Esbjerg.

Primary investigator: Torben Tranborg Jensen, MD.

Patients will be randomized 1:1 to one of the two treatment arms:

1. **Intervention group**: intravenous beta-lactam in combination with oral ciprofloxacin for 14 days
2. **Control group**: no antibiotic treatment

The analyses described in this document will be performed by the coordinating investigator, Josefin Eklöf, in cooperation with the scientific sponsor, Jens Ulrik Stæhr Jensen, once the data have been entered, cleaned, and released for use.

This statistical analysis plan provides a detailed description of the statistical analyses that will be performed for the evaluation of the primary and secondary endpoints of the TARGET-ABC study.

The analyses described in this document are compatible with the recommendations of the CONSORT 2010 statement.

**Analyses:**

Data will be analysed using intention-to-treat (ITT) principles, including all the data available, regardless of whether the participant completed the intervention or not. The aim of the ITT analysis is also to provide unbiased comparisons among the two study groups and to avoid the effects of potential study dropouts and protocol deviations. The primary outcome will also be subject to a modified ITT analysis (in study participants who started but did not complete the intervention) and per protocol analysis (in study participants who

completed intervention).

A Consort diagram of participants will be presented in the study.

Patients who withdraw their consent for the use of their data will not be included in any analysis. Patients who merely withdraw consent to the intervention, will be included in the ITT and modified ITT analysis. We will report cases of withdrawal and the study group to which the participant was originally allocated.

All analyses will be performed using SAS software.

**Sample size:**

The power to avoid type II error is 80% (1-β) at a two-sided 5% significance level. We used a group-sequential design, allowing for one interim analysis at half target recruitment. This provides a sample size of 150 subjects. All confidence intervals reported will be 95% confidence intervals.

**Descriptive analyses:**

The following baseline characteristics will be presented within each randomized study group:

- Age, years, median (IQR)
- Male sex, n (%)
- Ethnicity (Caucasian, African (incl. Afro-American), Asian, Inuit, Unknown/other), n (%)
- Body mass index (kg/m2), median (IQR)
- Medical Research Council dyspnea scale, n (%)
- Current smoking, n (%)
- Former smoking, n (%)
- Non-smoking, n (%)
- Pack-years tobacco history, median (IQR)
- COPD assessment test score (CAT), median (IQR)
- Support with activities of daily living at home, n (%)
- Increased dyspnea, n (%)
- Increased sputum volume, n (%)
- Increased sputum purulence, n (%)
- Increased cough, n (%)
- Systolic blood pressure (mm Hg), median (IQR)
- Diastolic blood pressure (mm Hg), median (IQR)
- Heart rate, beats/min, median (IQR)
- Oxygen saturation with nasal oxygen, median (IQR)
- Respiratory rate, breaths/min, median (OQR)
- Temperature (°C), median (IQR)
- Exacerbation frequency in previous year, median (IQR)
- Current or former use of respiratory medication, including antibiotics, n (%)
- Use of long-term oxygen therapy, n (%)
- Use of noninvasive mechanical ventilation, n (%)
- Co-morbidities, n (%)
- FEV1, L, median (IQR)
- FEV1 % predicted, median (IQR)
- FVC, L, median (IQR)
- FVC % predicted, median (IQR)
- FEV1/FVC ratio, %, median (IQR)

**Follow-up data /missing data**

We expect the extent of missing data to be small in the current trial, and we do not expect that any patients will be lost to follow-up for the primary endpoint.

Following measures will be used in case of missing data:

1. For each baseline variable, the percent of any missing values will be reported.
2. The proportion of patients followed for each outcome data parameter will be reported for the predefined primary and secondary outcomes – as well as in any potential exploratory outcome analyses suggested by external reviewers or editors.
3. Characterization participants for whom no outcomes were observed.
4. Report possible reasons for missing outcome data.
5. We will perform sensitivity analysis to quantify the effect of missing outcome data using multiple imputation on study results

**Primary objective and outcomes**

The primary outcome is

1. “days alive and without exacerbation from day 20 to day 365 from randomization”, defined as the time alive and without exacerbation between day 20-365 from the date of recruitment.
2. “time to prednisolone and/or antibiotic requiring exacerbation or death, in primary or secondary health care sector, from day 20 to day 365 from randomization”, defined as time to exacerbation or death between day 20-365 from the date of recruitment.

Data for the primary outcome analyses will be analyzed separately and presented as mean [95%CI] and corresponding t-test and additionally for sensitivity analysis median [IQR] with corresponding non-parametric test, e.g. Mann-Whitney U-test.

The primary outcome will also be analyzed as an adjusted analysis using a multivariable Cox proportional hazards model, adjusting for the following variables: sex (male vs. female), CAT-score at recruitment (< 21 vs. ≥ 21) and FEV1 % predicted at recruitment (<50% vs. >= 50%).

**Secondary objective and outcomes**

The secondary objective is to determine whether the clinical outcome for patients receiving anti-pseudomonal antibiotics will be more favorable compared to no antibiotic treatment, and to explore the microbiological cure to the antibiotic intervention.

1. Death within 365 days from randomization.

Analysis: Fisher's exact test or Chi squared test.

1. Number of re-admissions with pulmonary exacerbation within 365 days from randomization.

Analysis: Fisher's exact test or Chi squared test.

1. Number of days with non-invasive-ventilation or invasive ventilation within 90 days from randomization.

Analysis: Fisher's exact test or Chi squared test.

1. For patients with COPD: days alive and out of hospital within day 20-365 from randomization. Analysis: T-test as well as non-parametric test, e.g. Mann-Whitney U-test.
2. Microbiological cure (defined as *P. aeruginosa*-negative sputum culture until day 90).

Analysis: Fisher's exact test or Chi squared test.

1. Clinical cure day 14 (defined as cessation or improvement of clinical signs and symptoms related to *P. aeruginosa* before or on day 14).

Analysis: Fisher's exact test or Chi squared test.

1. Change in COPD Assessment Test (CAT) from randomization to day 90.

Analysis: ANOVA will be used to analyse the difference between the two means.

1. Change in body mass index (BMI) from randomization to day 90.

Analysis: ANOVA will be used to analyse the difference between the two means.

1. Change in FEV_1_ from randomization to day 90.

Analysis: ANOVA will be used to analyse the difference between the two means.

1. Decrease of ≥ 200 ml in FEV_1_ from randomization to day 365.

Analysis: Fisher's exact test or Chi squared test.

**Interim Analysis:**

The interim analysis will focus on reporting:

1. Baseline characteristics
2. Primary outcome: Days alive and without exacerbation from day 20 to day 365 from randomization (using O- Brien-Fleming Plot)
3. Primary outcome: Time to prednisolone and/or antibiotic requiring exacerbation or death, in primary or secondary health care sector, from day 20 to day 365 from randomization (using O- Brien-Fleming Plot)
4. All-cause mortality at 365 days
5. Microbiological cure
6. Futility assessment. Recruitment rate compared to planned recruitment.

**Blinding of the statistician**

The detailed analysis plan was written in strict concordance with the trial protocol approved by the regulatory authorities prior to recruitment initiation. The entire statistical analysis plan is published at www.coptrin.dk (before the trial was finalized and while the database was closed). All analyses will be done prior to breaking of the randomization code (analysis comparisons between “arm A” and “arm B”). The coordinating investigator and the study sponsor and principal investigator will conjointly perform all the data analyses according to this plan, except the interim analysis, which will be performed by a statistician who is not an investigator in the trial. An unblinding date will be chosen and published online at www.coptrin.dk and on this date, the allocation will be unblinded. After the unblinding, no further analysis will not be done, except on demand of reviewer or editor during the publication process.

**Figures and tables**

The first figure will be a Consolidated Standards of Reporting of Randomized Trials (CONSORT) flow chart. The second figure will be a Kaplan-Meier plot to describe the process of death by treatment arms. The first table will be the baseline characteristics of the ITT population. The second table will be of the primary and secondary outcomes according to the two groups and pair-wise comparisons.
